# Influence of cancellous bone microstructure on ultrasonic attenuation: a theoretical prediction

**DOI:** 10.1186/s12938-019-0724-4

**Published:** 2019-10-25

**Authors:** Jinjin Liu, Li Lan, Jiafeng Zhou, Yunjun Yang

**Affiliations:** 1 Department of Radiology, The First Affiliated Hospital of Wenzhou Medical University, Ouhai Qu, Wenzhou, 325000 Zhejiang China; 2 Department of Ultrasonography, The First Affiliated Hospital of Wenzhou Medical University, Wenzhou, Zhejiang China

**Keywords:** Cancellous bone, Microstructure, Ultrasound attenuation, Scattering, Absorption, Quantitative ultrasound

## Abstract

**Background:**

Quantitative ultrasound has been used for the assessment of cancellous bone status. The attenuation mechanisms of cancellous bone, however, have not been well understood, because the microstructure of cancellous bone is significantly inhomogeneous and the interaction between ultrasound and the microstructure of cancellous bone is complex. In this study, a theoretical approach was applied to investigate the influence of the microstructure of cancellous bone on ultrasonic attenuation.

**Results:**

The scattering from a trabecular cylinder was significantly angle dependent. The dependencies of the ultrasonic attenuation on frequency, scatterer size, and porosity were explored from the theoretical calculation. Prediction results showed that the ultrasonic attenuation increased with the increase of frequency and decreased linearly with the increase in porosity, and the broadband ultrasound attenuation decreased with the increase in porosity. All these predicted trends were consistent with published experimental data. In addition, our model successfully explained the principle of broadband ultrasound attenuation measurement (i.e., the attenuation over the frequency range 0.3–0.65 MHz was approximately linearly proportional to frequency) by considering the contributions of scattering and absorption to attenuation.

**Conclusion:**

The proposed theoretical model may be a potentially valuable tool for understanding the interaction of ultrasound with cancellous bone.

## Background

As a major public health problem, osteoporosis is a metabolic skeletal disease where the risk of fragility fracture increases because the bone becomes porous and brittle [1]; it is the most common reason for a broken bone among the elderly [2]. Dual-energy X-ray absorptiometry is considered the gold standard for bone status assessments [3]; however, quantitative ultrasound may be a favorable tool for community-based screening of the high-risk population because it is a cheap, easy-to-access and non-ionizing method [4–6]. One of the quantitative ultrasound parameters is ultrasonic attenuation, which has been reported both in vitro and in vivo [4, 7, 8]. Bone mineral reduction is the characteristic of osteoporosis, and previous research indicated that the bone mineral content was associated with ultrasonic attenuation [4, 9]. Langton and colleagues [4] found a quasi-linear dependence of attenuation on frequency on human cancellous bone over the approximate frequency range of 0.3–0.65 MHz; based on this finding, the broadband ultrasound attenuation, which is the slope of the ultrasonic attenuation coefficient with respect to frequency, has received great attention to assess the bone status [4, 10]. Although the broadband ultrasound attenuation has been successfully applied to evaluate the bone status, the attenuation mechanisms of ultrasound propagation in bone are still not well understood, especially for cancellous bone.

The overall attenuation is the combination of absorption and scattering. Cancellous bone is a porous composite material composed of marrow and trabecular networks [11, 12]. Marrow is a fluid-like viscous medium; the ultrasound energy will be absorbed due to its viscous material properties. Compared with marrow, trabeculae have much higher acoustic impedance ($7.9 \times 10^{6}$ vs $1.3 \times 10^{6} {\text{kg}}/\left( {{\text{s}}\;{\text{m}}^{2} } \right)$ for common values); moreover, the complex microstructure of cancellous bone, such as the size of trabeculae, bone volume fraction, and orientations of trabecular network, significantly influences the ultrasonic propagation in cancellous bone [13]. The large mismatch of acoustic impedance and complex microstructure of cancellous bone may lead to strong scattering of an incident ultrasonic wave. Therefore, both the absorption and scattering may play important roles as ultrasonic waves propagate in cancellous bone; however, few theoretical models consider energy loss from both scattering and absorption.

Biot’s theory [14, 15], which predicts that absorption is due to viscous losses at internal interfaces, has been applied to cancellous bone [16]. Numerous parameters are required to be known in this theory, and generally these parameters are difficult to be acquired, which limits its application in cancellous bone. In addition, this theory underestimates the experimentally observed attenuation in cancellous bone [17]. Meanwhile, some other scholars suggested that scattering might be a crucial contribution of attenuation for cancellous bone besides the absorption. Chaffai et al. [18] used the weak scattering model, which was based on a random description of the scattering tissue. Strelitzki et al. [19] adopted the binary mixture model, which was based on the assumption that the scattering was proportional to the mean fluctuations of velocity and density. The density fluctuation was ignored in their binary mixture model for cancellous bone. Although the weak scattering model and the binary mixture model have been successfully applied to model scattering from soft tissue, they require isotropy and small contrasts in acoustic properties between the solid trabeculae and the fluid marrow, which is not satisfied because of the large mismatch of acoustic impedance between trabeculae and marrow. Wear [20] proposed the Faran cylinder model, which assumed that trabecular cylinders with diameters much smaller than incident wavelength were the dominant source of scattering in bone. He further suggested that absorption was likely to be significant component of attenuation than scattering because of the discrepancy of attenuation at diagnostic frequencies between the theoretical prediction and experiment data. Thus, to well understand the attenuation mechanisms of cancellous bone, it is necessary to consider the energy loss from both the absorption and scattering attenuation mechanisms. The objective of this article was to investigate the dependence of ultrasonic attenuation on the cancellous bone microstructure by considering both absorption and scattering from a theoretical prediction.

## Results

**Fig. 1:**
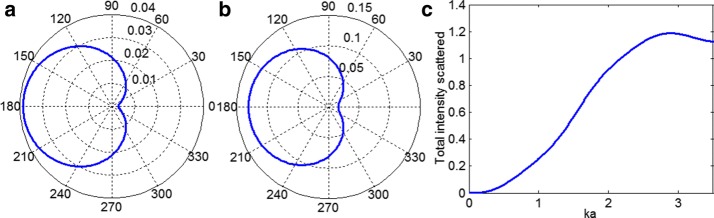
Polar diagrams showing the distribution in angle of the intensity of the scattered wave for **a** $\lambda = 8\pi a$ and **b** $\lambda = 4\pi a$; **c** the dependence of the total scattered intensity on $ka$

Figure 1a, b shows typical scattering patterns of the scattered wave from a trabecular cylinder at wavelength $\lambda = 8\pi a$ and $\lambda = 4\pi a$. The scattering was significantly angle dependent; the wave was scattered almost uniformly in the backward directions, and the intensity amplitude of scattered wave was much smaller in the forward direction than that in the backward direction. Figure 1c demonstrates the normalized quantity $\gamma^{\text{sca}}$, which represents the total scattered intensity, as a function of $ka = 2\pi a/\lambda$. The total intensity scattered varied nonlinearly with $ka$.

**Fig. 2:**
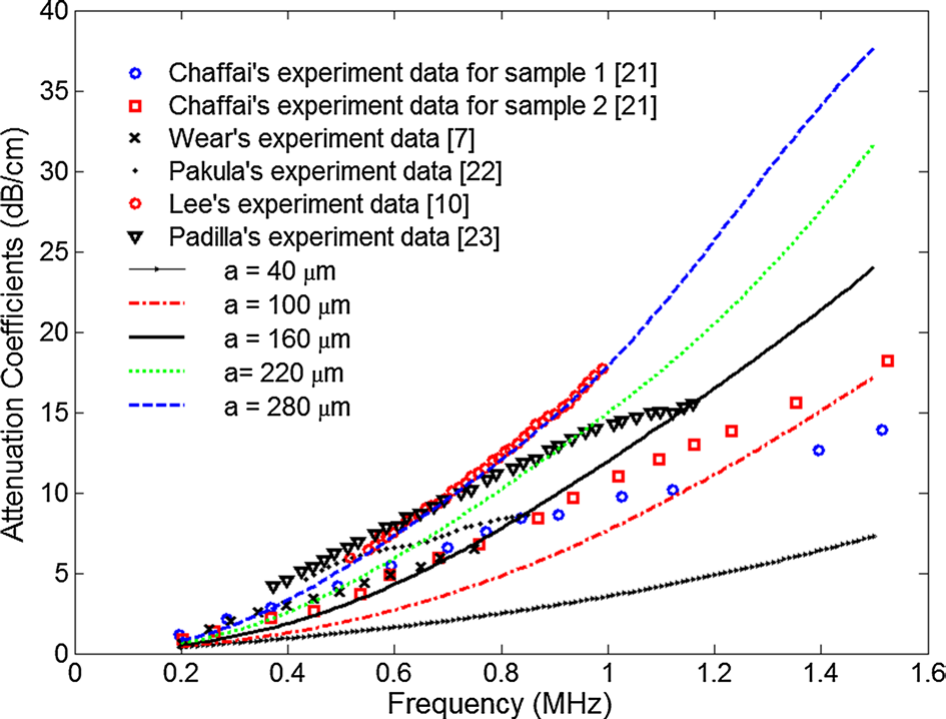
Overall attenuation coefficient as a function of frequency for different trabecular size

Figure 2 shows the attenuation coefficients as a function of frequency for different trabecular sizes at the porosity of 80%. Six experiment data from literature were also plotted in the figure. It was noted that the attenuation increased with the increase in frequency, and this trend was consistent with previous experimental reports [7, 10, 21–23]. Theoretical calculation also revealed that the attenuation was higher for larger trabecular size.

**Fig. 3:**
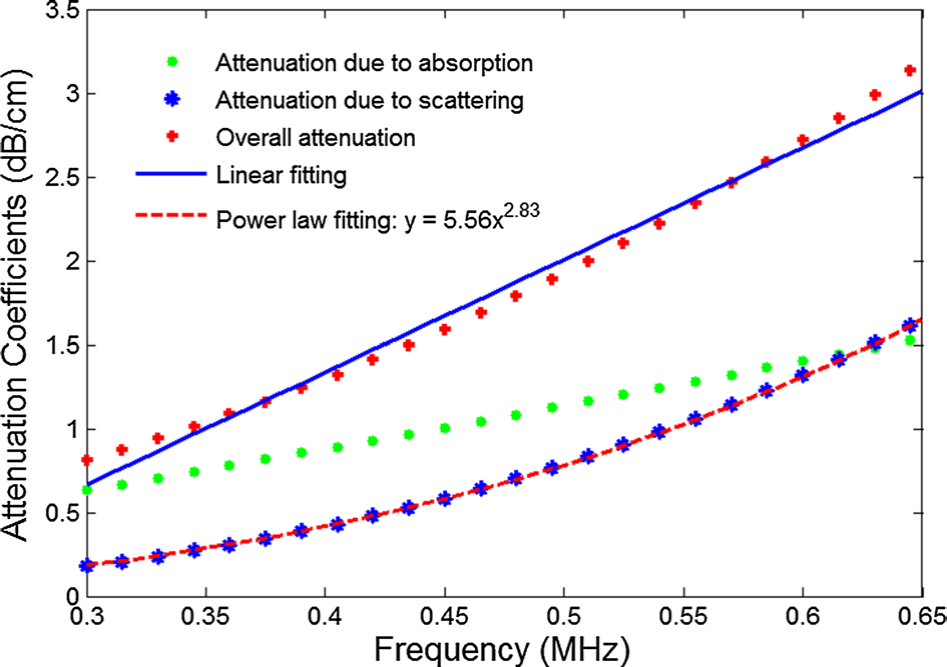
Attenuation due to absorption and scattering as a function of frequency at a porosity of 80% and a trabecular size of 100 µm

Figure 3 demonstrates the attenuations due to absorption and scattering over the frequency range of 0.3–0.65 MHz at a trabecular size of 100 µm and a porosity of 80%. Both attenuation due to absorption and scattering increased as the increasing of frequency. The attenuation was greater due to absorption than due to scattering below the frequency of 0.6 MHz; however, the attenuation due to scattering, which grew in the form of a power law function, increased faster than that due to absorption, which grew approximately linearly. It was noted that the power exponent was found to be 2.83 (close to 3.0) by fitting the attenuation coefficient due to scattering with a power law function. Although the attenuation from scattering was strongly non-linear, the overall attenuation approximately linearly increased with the frequency, which might be mainly caused by the compensation of the attenuation from absorption.

**Fig. 4:**
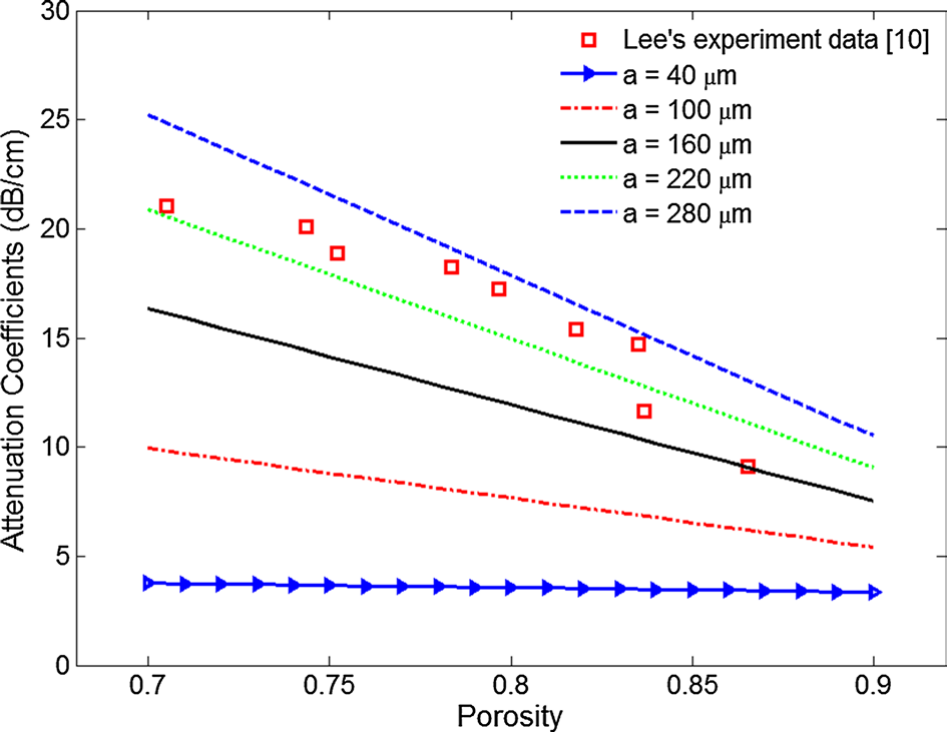
Attenuation coefficient as a function of porosity at different trabecular sizes

**Fig. 5:**
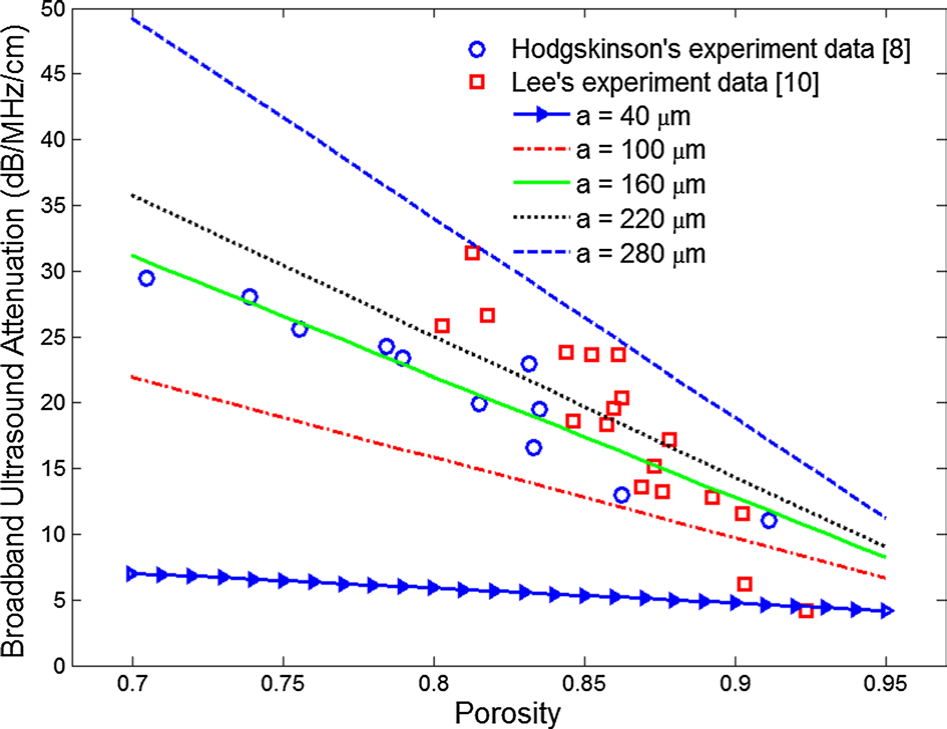
Broadband ultrasound attenuation as a function of porosity at different trabecular sizes

The theoretical attenuation coefficients versus porosity at different trabecular sizes are shown in Fig. 4. The ultrasound attenuation coefficient decreased linearly with the increase in porosity, and a similar trend was observed from Lee’s experiment data [10]. The broadband ultrasound attenuation coefficient at different trabecular sizes is illustrated in Fig. 5. It was observed that the broadband ultrasound attenuation coefficient decreased with the increase in porosity from the theoretical calculation, and a similar trend was found from Hdgskinson’s and Lee’s experimental studies [8, 10].

## Discussion

In this study, a theoretical model was applied to investigate the influence of microstructure of cancellous bone on ultrasonic attenuation by considering attenuation from both scattering and absorption. This model did not fully account for the trabecular microstructure; however, it successfully predicted the trends between ultrasonic attenuation coefficient and trabecular size, bone volume fraction, and frequency. The dependence of broadband ultrasound attenuation on porosity was also successfully revealed.

Experimental studies have shown that the attenuation coefficient is a quasi-linear function of frequency over the typical range of 0.3–0.65 MHz [4, 24, 25], and this quasi-linear assumption is the basis of broadband ultrasound attenuation measurement. From the perspective of scattering, however, the quasi-linear assumption is difficult to be explained. As in the low-frequency limit, the scattered intensity from a cylinder is proportional to the frequency cubed [26]. Strong non-linear scattering would occur. Wear [20] investigated the frequency dependence of ultrasonic backscatter from human cancellous bone. He observed the discrepancy of attenuation at diagnostic frequencies between the theoretical prediction and experimental data, and then proposed that the absorption was likely to contribute more to attenuation than scattering in the low-frequency range. In our calculation, we found that attenuation due to scattering was well fitted by a power law function with a power index very close to 3. Although the scattering portion of the intensity was almost frequency-cubed dependent, we found that the attenuation due to absorption portion was comparable to the attenuation due to scattering and was more linear in trend. The non-linear trend of attenuation due to scattering was compensated by the introduction of absorption, which caused the quasi-linear behavior of the overall attenuation. Good agreement between the theoretical prediction and linear fitting was observed in the frequency range of 0.3 to 0.65 MHz. Thus, the approach presented here theoretically validates the quasi-linear assumption of attenuation coefficient for cancellous bone over a typical frequency range of 0.3–0.65 MHz.

There are several limitations in our approach. First, the trabeculae were assumed to be cylinders with uniform radius in the model, and the geometrical heterogeneity of trabecular structure was not fully accounted for. This simple assumption may influence the accuracy of our theoretical predictions and further hinder the clinical application. The real shape, dimension, and orientation of trabeculae are highly complex. Future studies are required to obtain a more exact prediction of attenuation from the cancellous bone using more realistic bone microstructure. Cancellous bone may be considered as a composite material composed of matrix and inclusions. In the field of micromechanics, the ellipsoidal is used to define the inclusion shape and the orientation distribution function is used to define the orientation of inclusion in the matrix [27, 28], which may be applied to characterize the microstructure of cancellous bone. Second, multiple scattering was ignored in this study and only single scattering was considered. However, our assumption may be appropriate, since Wear [20] experimentally investigated the frequency dependence of ultrasonic backscatter from human trabecular bone and suggested that multiple scattering effects on the average were insignificant. Despite these limitations, the model successfully predicted the trends of ultrasonic attenuation in terms of frequency, trabecular size, and porosity, and provided a theoretical explanation for the quasi-linear assumption of attenuation coefficient for cancellous bone over a typical frequency range of 0.3–0.65 MHz. It is expected that this model may be a potentially valuable tool for understanding the interaction of ultrasound with bone.

## Conclusions

In conclusion, both absorption and scattering were considered for the prediction of ultrasonic attenuation of cancellous bone. The model successfully explained the quasi-linear behavior of the attenuation coefficient at low frequencies. The dependency of the ultrasonic attenuation on frequency, scatter size, and porosity was also predicted, and corresponding trends were consistent with previous reports. It is expected that our results may provide a valuable insight into ultrasonic propagation in human cancellous bone and aid in the bone assessment for the diagnosis of osteoporosis in clinics.

## Methods

A theoretical method was adopted from the literature [29] in which ultrasonic attenuation coefficient was assumed to be the summation of absorption and scattering from both the matrix and inclusions. This theory models the independent scattering and wave attenuation in two-phase composites and takes into consideration the attenuation property of the matrix and the scattering and absorption cross sections of a single inclusion. The exact formulation for the overall attenuation coefficient is given by

1 $$\alpha = \left( {1 - \emptyset } \right)\alpha_{1} + 0.5n_{\text{s}} \left( {\gamma^{\text{sca}} + \gamma^{\text{abs}} } \right),$$

where $\alpha$ is the total attenuation coefficient, $\emptyset$ is the inclusion volume fraction, $\alpha_{1}$ is the attenuation coefficient of matrix, $n_{\text{s}}$ is the number density of inclusions, $\gamma^{\text{sca}}$ is the scattering cross section of a single inclusion in the infinite matrix, and $\gamma^{\text{abs}}$ is the absorption cross section of a single inclusion in the infinite matrix.

In this study, the cancellous bone was modeled as a two-phase composite material with inclusions of identical trabecular cylinders with radius $a$ embedded in the homogeneous matrix of marrow. The typical trabecular thickness is approximately in the range of 80 to 440 µm [30–33]. Moreover, the trabecular cylinders were assumed to be long relative to the ultrasound beam cross section and perpendicular to the incident ultrasonic wave. The microstructure of cancellous bone in this model was consistent with one type of microstructures of cancellous bone [20, 34], and only rod shaped trabeculae were used. The absorption part from the trabecular cylinders was ignored due to small bone volume fraction and large difference of impedance in solid trabecular and fluid marrow. Under these assumptions, the total ultrasonic attenuation coefficient of cancellous bone can be written as

2 $$\alpha = \left( {1 - \emptyset } \right)\alpha_{1} + \frac{1}{2}\frac{\emptyset }{{\pi a^{2} }}\gamma^{\text{sca}} ,$$

where $a$ is the radius of trabecular cylinder and $\emptyset$ is the bone volume fraction. The value of $\left( {1 - \emptyset } \right)$ is defined as the porosity. The attenuation coefficient of marrow was determined using a power law equation as follows:

3 $$\alpha_{1} = a_{0} f^{n} ,$$

where $f$ is the frequency in MHz, $a_{0}$ and $n$ were determined to be 3.15 and 1.15 by fitting Gammel’s experimental data [35], respectively.

The scattering cross section of an ultrasound wave scattered from a solid cylinder, $\gamma^{\text{sca}}$, is given by [36]

4 $${\gamma^{\text{sca}}} = \frac{{{\varPi_{\text{s}}}}}{I} = \frac{4a}{ka}\mathop \sum \limits_{m = 0}^\propto {\epsilon_m}{\sin^2}\left( {\eta_m} \right),$$

where $$\varPi_{\text{s}}$$ is the total power scattered by the cylinder per unit length, $$I$$ is the incident intensity, $$k$$ is the wavenumber defined by $$2\pi /\lambda$$ ($$\lambda$$ is the wavelength in the marrow surrounding the trabecular), $${\varepsilon_m}$$ is the Newman constants ($${\varepsilon_0} = 1$$ and $${\varepsilon_m} = 2$$ for $$m > 0)$$, and $$\eta_{m}$$ is the phase-shift angle of the *n*th scattered wave. $$\eta_{m}$$ can be calculated from [37]

5 $$\tan \eta_{m} = \tan \delta_{m} \left( x \right)\left[ {\tan \varPhi_{m} + \tan \alpha_{m} \left( x \right)} \right]/\left[ {\tan \varPhi_{m} + \tan \alpha_{m} \left( x \right)} \right],$$

where

6 $$x = ka,$$

7 $$\delta_{m} = \tan^{ - 1} \left[ { - J_{m} \left( x \right)/N_{m} \left( x \right)} \right],$$

8 $$\alpha_{m} \left( x \right) = \tan^{ - 1} \left[ { - xJ_{m}^{\prime } \left( x \right)/J_{m} \left( x \right)} \right],$$

9 $$\beta_{\text{m}} \left( x \right) = \tan^{ - 1} \left[ { - xN_{\text{m}}^{\prime } \left( x \right)/N_{\text{m}} \left( x \right)} \right],$$

and

10 $$\tan \varPhi_{m} = \left( {\rho /\rho_{1} } \right)\tan \xi_{m} \left( {x_{1} ,\nu } \right),$$

where $J_{m}$ and $N_{m}$ correspond to Bessel and Newman functions, respectively; $\rho$ and $\rho_{1}$ are the density of marrow and trabecular, respectively, and $\upsilon$ is the Poisson’s ratio of trabecular bone. The angle $\xi_{m} \left( {x_{1} ,\nu } \right)$ is given by

11 $$\xi_{m} \left( {x_{1} ,\nu } \right) = \tan^{ - 1} \left[ { - \frac{{x_{2}^{2} }}{2}\frac{{\frac{{\tan \alpha_{m} \left( {x_{1} } \right)}}{{\tan \alpha_{m} \left( {x_{1} } \right) + 1}} - \frac{{m^{2} }}{{m^{2} + m - 1 - 0.5x_{2}^{2} + \tan \alpha_{m} \left( {x_{2} } \right)}}}}{{\frac{{m^{2} - 0.5x_{2}^{2} + 2\tan \alpha_{m} \left( {x_{1} } \right)}}{{\tan \alpha_{m} \left( {x_{1} } \right) + 1}} - \frac{{m^{2} \left[ {\tan \alpha_{m} \left( {x_{2} } \right) + 1} \right]}}{{m^{2} - 0.5x_{2}^{2} + \tan \alpha_{m} \left( {x_{2} } \right)}}}}} \right],$$

where $x_{1} = k_{1} a = \frac{\omega }{{c_{1} }}a$, $x_{2} = k_{2} a = \frac{\omega }{{c_{2} }}a$, and $c_{1}$ and $c_{2}$ correspond to the longitudinal and shear wave velocity in the trabeculae, respectively.

Substitution of Eqs. (5)–(11) into Eq. (4) yields the scattering cross section of an ultrasound wave scattered from a solid cylinder, $\gamma^{\text{sca}} .$ The overall ultrasonic attenuation coefficient of the cancellous bone, $\alpha ,$ can be calculated by further substitution of Eqs. (3) and (4) into Eq. (2). It can be observed from the above formulations that the attenuation coefficients of cancellous bone depend on the incident frequency, the material properties of the matrix and inclusions, and volume fraction and geometry of the inclusions. For very long wavelength (i.e., value of $ka$ goes to zero), the scattering cross section can be simply written as

12 $$\gamma^{\text{sca}} = 3/4\pi^{2} (ka)^{3} .$$

In the following section, we will apply these formulations to predict the influence of frequency, scatter size, and bone volume fraction on the ultrasonic attenuation of cancellous bone. In the calculation, the values of density, longitudinal wave speed, and the Poison’s ratio of solid trabeculae were adopted from literature [38, 39] and set to 2000 kg/m^3^, 3929 m/s, and 0.24, respectively. The density and wave speed in the marrow were assumed to be 900  kg/m^3^ and 1400 m/s, respectively [40].

## Data Availability

Not applicable.
